# Mutations of *pvdhfr* and *pvdhps* genes in *vivax* endemic-malaria areas in Kota Marudu and Kalabakan, Sabah

**DOI:** 10.1186/s12936-016-1109-9

**Published:** 2016-02-05

**Authors:** Umi Rubiah Sastu, Noor Rain Abdullah, Nor Azrina Norahmad, Muhammad Nor Farhan Saat, Prem Kumar Muniandy, Jenarun Jelip, Moizin Tikuson, Norsalleh Yusof, Hasidah Mohd Sidek

**Affiliations:** Herbal Medicine Research Centre, Institute for Medical Research, Ministry of Health, Jalan Pahang, 50588 Kuala Lumpur, Malaysia; Sabah State Health Department, Level 3, Rumah Persekutuan, Jalan Mat Salleh, 88590 Kota Kinabalu, Sabah Malaysia; District Health Office Kota Marudu, PO Box 421, 89108 Kota Marudu, Sabah Malaysia; Faculty of Science and Technology, School of Bioscience and Biotechnology, Universiti Kebangsaan Malaysia, 43600 Bangi, Malaysia

**Keywords:** *Plasmodium vivax*, Molecular marker, Sulfadoxine-pyrimethamine, Dihydrofolate reductase *(dhfr)* gene, Dihydropteroate synthase *(dhps)* gen

## Abstract

**Background:**

Malaria cases persist in some remote areas in Sabah and Sarawak despite the ongoing and largely successful malaria control programme conducted by the Vector Borne Disease Control Programme, Ministry Of Health, Malaysia. Point mutations in the genes that encode the two enzymes involved in the folate biosynthesis pathway, dihydrofolate reductase (DHFR) and dihydropteroate synthase (DHPS) enzymes confer resistance to pyrimethamine and sulfadoxine respectively, in both *Plasmodium falciparum* and *P. vivax*. The aim of the current study was to determine the mutation on both *pvdhfr* at codon 13, 33, 57, 58, 61, 117, and 173 and *pvdhps* genes at codon 383 and 553, which are potentially associated with resistance to pyrimethamine and sulfadoxine in *P. vivax* samples in Sabah.

**Methods:**

Every individual was screened for presence of malaria infection using a commercial rapid dipstick assay, ParaMax-3™ (Zephyr Biomedical, India). Individuals tested positive for *P. vivax* had blood collected and parasite DNA extracted. The *pvdhfr* and *pvdhps* genes were amplified by nested-PCR. Restriction fragment length polymorphism (RFLP) was carried out for detection of specific mutations in *pvdhfr* at codons 13Leu, 33Leu, 57Ile/Leu, 58Arg, 61Met, 117Asn/Thr, and 173Leu and *pvdhps* at codons 383Gly and 553Gly. The PCR–RFLP products were analysed using the Agilent 2100 Bioanalyzer (Agilent Technology, AS).

**Results:**

A total of 619 and 2119 individuals from Kalabakan and Kota Marudu, respectively participated in the study. In Kalabakan and Kota Marudu, 9.37 and 2.45 % were tested positive for malaria and the positivity for *P. vivax* infection was 4.2 and 0.52 %, respectively. No mutation was observed at codon 13, 33 and 173 on *pvdhfr* and at codon 553 on *pvdhps* gene on samples from Kalabakan and Kota Marudu. One-hundred per cent mutations on *pvdhfr* were at 57Leu and 117Thr. Mutation at 58Arg and 61Met was observed to be higher in Kota Marudu 72.73 %. Mutation at 383Gly on *pvdhps* was highest in Kalabakan with 80.77 %. There are four distinct haplotypes of *pvdhfr*/*pvdhps* combination.

**Conclusions:**

The presence of triple and quintuple mutation combination suggest that the *P. vivax* isolates exhibit a high degree of resistant to sulfadoxine, pyrimethamine and sulfadoxine-pyrimethamine combination therapy.

## Background

Malaysia is pursuing malaria elimination and has a national goal to eliminate malaria by 2020, and categorizes as in pre-elimination phase by the World Health Organization [1]. In Malaysia, malaria cases showed a decreased in 2010, 2011, 2012, and 2013, but slightly increased in 2014; incidents of malaria were 6650, 5306, 4725, 3850, and 3923, respectively [2]. Malaria is endemic in Sabah and most of the malaria cases occurs in remote areas of the interior region of Sabah or those areas along the borders of Malaysia-Indonesia, where mosquito vectors and infected individuals are present. Inadequate transport and communication facilities to these areas are a problem for access to diagnosis and treatment and pose challenges for control programmes. Sabah shares borders with North Kalimantan of Indonesia in the south, facing Sebatik Island, partly within Malaysia and partly within Indonesia. This geographic location, together with population movement, could facilitate malaria transmission in the country.

Since 2006, malaria cases in Malaysia were mostly attributed to *Plasmodium vivax,* which contributed to more than 50 % of malaria infection, followed by *P. falciparum*. However in 2014, the trend changed, when *P. knowlesi* infection was recorded in the highest number of cases, 65.9 % of all malaria cases in Malaysia, followed by *P. vivax* and *P. falciparum*, 18.6 and 9.2 %, respectively. Most human malaria and zoonotic malaria cases in Malaysia come from Sabah, followed by Sarawak [2].

*Plasmodium vivax* was considered to cause benign and uncomplicated cause of illness, but with new molecular tools, it is evident that *P. vivax* parasite infection could also be involved in multiple organ dysfunction and cause of severe malaria similar to *P. falciparum* in adults and children, including infants [3, 4].

According to the Malaysia National Antibiotic Guideline 2008, the treatment for vivax malaria is chloroquine and primaquine. However, for mixed infection cases (co-infection of *P. falciparum* and *P. vivax*), the treatment is similar to *P. falciparum* [5]. In the past, chloroquine (CQ) was the first-line anti-malarial drug used for the treatment of malaria. Sulfadoxine-pyrimethamine (SP), also known as Fansidar^®^, was used in areas with suspected CQ-resistance and for the treatment of mixed infection cases. Mixed infection of *P. falciparum* and *P. vivax* is frequent and thus the use of SP is likely to have exposed *P. vivax* to this drug. The use of SP in the treatment of uncomplicated falciparum malaria started in 1976. By 1996 resistance to SP was observed to be widely distributed in Peninsular Malaysia [6]. Additionally, SP combination is still occasionally used as presumptive treatment for individuals suspected of malaria in remote areas where diagnosis and treatment is not accessible. Therefore, *P. vivax* populations are often exposed to SP pressure and this might have caused the selection of the SP-resistant allele in *P. vivax* isolates [7].

Molecular epidemiology studies have shown that the point mutations in the genes that encode the two enzymes involved in the folate biosynthesis pathway, dihydrofolate reductase (DHFR) and dihydropteroate synthase (DHPS) enzymes confer resistance to pyrimethamine and sulfadoxine, respectively, in both laboratory [8] and in natural parasite populations, in both *P. falciparum* and *P. vivax* parasites [9, 10]. Molecular studies have identified more than 20 different alleles for *pvdhfr*, of which mutations at codon 57Leu, 58Arg, 61Met, 117Thr/Asn, and 173Ile have been reported to be involved in clinical antifolate resistance. The presence of double mutation at codon 58Arg and 117Thr and triple mutation at codon 57Leu, 58Arg and 117Thr at *pvdhfr* gene are associated with delayed parasite clearance following SP treatment [11]. Quadruple mutations at 57Leu, 58Arg, 61Met, and 117Thr in this gene corresponded to therapeutic failure of SP treatment among *P. vivax*-infected patients [12, 13]. Five mutations have been identified on the *pvdhps* gene at codons 382, 383, 442, 512, 553, and 585. Mutation at codon 382Ala/Cys, 383Gly, 512Met/Thr/Glu, and 553Gly of *pvdhps* is associated with sulfadoxine resistance [13, 14].

*Pvdhfr* mutant alleles of 58Arg and 117Thr/Asn were found in the southeast Asian countries of East Timor [15], Thailand [11, 16], Myanmar [16], Vietnam and Philippines [17], and India [18]. Mutation in *pvdhps* at amino acids 383Gly and 553Gly were highly prevalent in Thailand [19, 20]. In Malaysia, *P. falciparumdhfr* and *dhps* genes have been studied extensively in endemic areas of Sabah, the villages in the districts of Kalabakan, Tawau bordering to North Kalimantan [21], the interior part of Sabah, Keningau, and Nabawan [22]. Both studies have indicated the high prevalence of mutation in *pfdhfr* and *pfdhps* conferring resistance to SP, which could predict treatment failure.

The aim of the current study was to investigate the frequency of mutation on both *pvdhfr* and *pvdhps* genes potentially associated with resistance to pyrimethamine and sulfadoxine in *P. vivax* field isolates in malaria-endemic areas of Sabah. This study will establish an epidemiological map of the distribution of SP-resistant vivax malaria in Sabah. The information obtained could contribute towards achieving the goal of the malaria elimination programme.

## Methods

### Study area

**Fig. 1:**
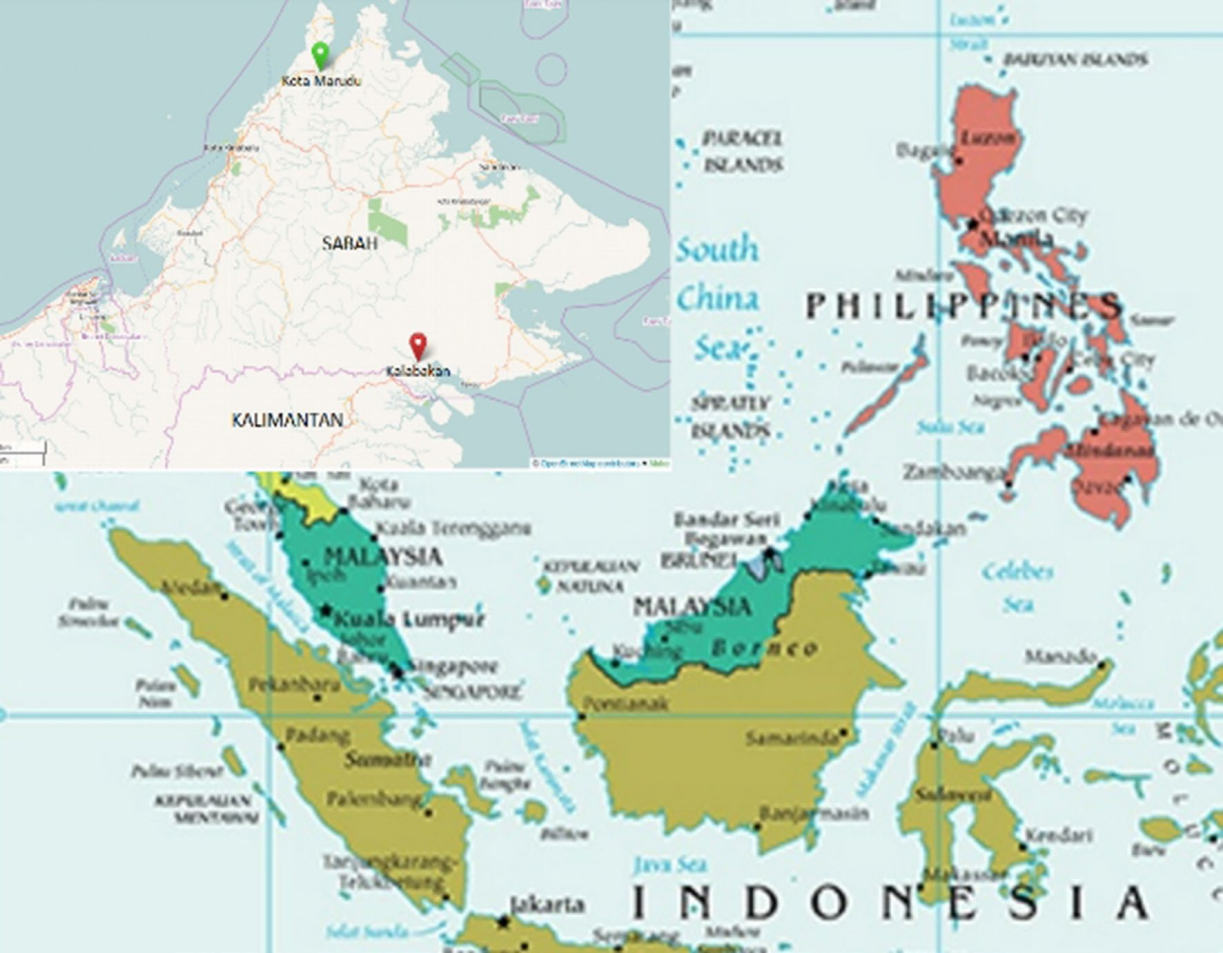
Map of Sabah showing the geographical sites where the 37 *P. vivax* isolates were collected: Kota Marudu (n = 11), Kalabakan (n = 26). In this study, samples were collected from two of Sabah divisions (illustrated in the *square* in top left corner), from Kota Marudu district in the Kudat Division and Kalabakan district in the Tawau Division. This map were generated by OpenStreetMap: http://www.openstreetmap.org/ and USGS National Map Viewer (public domain): http://viewer.nationalmap.gov/viewer/

*Plasmodium vivax* samples were collected from 23 villages in Kota Marudu, and 26 villages, road construction and loggers’ camps in Kalabakan, Sabah. Kalabakan is located 100 km from Tawau, towards the south of Sabah bordering Kalimantan, Indonesia while Kota Marudu is located in Kudat Division in the northern region of Sabah with 1083 km from Philippine (Fig. 1). The samples from Kalabakan were collected in 2008 and 2009 by active case detection. During those years, Kalabakan contributed to most of the malaria cases with 21.54 and 22.79 %, respectively, of the total cases in Sabah (Vector Borne Disease Control, 2008, 2009 unpublished data). Sample collections in Kota Marudu in 2011 and 2012 were by both active case detection and passive case detection. At the time the study was conducted, the number of malaria cases in Kota Marudu contributed 10 % of total cases in Sabah in 2011 (Vector Borne Disease Control, 2011 unpublished data).

### Sample collection

The study protocol was reviewed and approved by the Research Review Committee of the Institute for Medical Research (IMR) and Medical Research Ethics Committee (MREC), Ministry of Health Malaysia. For active case detection, the team visited the villages and the study was announced and briefed to all the population. Study information sheets were given to all who came to the briefing. All the population received the study information sheet, informed consent form and they were assisted whenever required in understanding the study. Individuals who consented to participate in the study were screened for malaria infection by finger-prick blood diagnosis in the field using rapid diagnosis test (RDT) kit (Paramax-3™, Zephyr Biomedicals, India). Some finger-prick blood was also used to prepare blood film for malaria parasite (BFMP) for determination of parasite density.

Individuals diagnosed positive for malaria infection by RDT had approximately 500 µl of blood collected by venipuncture and transferred to EDTA tubes. BFMP and blood in anticoagulant EDTA tubes were brought to the field station to be processed. The EDTA blood was spotted onto filter paper 3MM^®^ Whatman (Brentford, UK). The filter paper was allowed to dry completely by hanging on a wire line in the field station. After drying, they were packed into a small plastic zipper bags containing silica gels, labelled appropriately and transported to Institute for Medical Research for further molecular study. The BFMP were stained with Giemsa and examined for presence of malaria parasites by microscopy to determine the density of the infection. Individuals diagnosed positive with malaria were treated with a standard anti-malaria treatment by health clinic staff. The study managed to collect 58 samples from Kalabakan and 52 samples from Kota Marudu.

### DNA extraction and molecular diagnosis for confirmation of malaria species

Parasite DNA was extracted from blood spot filter paper by using QIAamp^®^ DNA Mini Kit (QIAmp; QIAGEN, Hilden, Germany) following the manufacturer’s instruction (Dried Blood Spot Protocol). The DNA was eluted with 150 µl AE Buffer (10 mM Tris–HCl pH 9.0 and 0.5 mM EDTA). The parasite DNA was used for molecular diagnosis of the malaria species and for genotyping studies. All samples underwent molecular speciation using Fuehrer et al. [23] for *P. falciparum*, *P. vivax* and *Plasmodium malariae*. All samples also underwent speciation for *P. knowlesi* using protocol from Imwong et al. [24]. Samples confirmed positive for *P. vivax* infection by the molecular diagnosis were included in the study.

### Amplification of *pvdhfr* and *pvdhps* gene

The *pvdhfr* and *pvdhps* genes were amplified from genomic DNA by nested-PCR. The amplifications of the *pvdhfr* was carried out as described previously [11, 25] with some modification on the final volume of the PCR reaction mix. In the amplification of *P. vivaxdhfr*-*ts* gene, size of 1876 bp, excluding the stop codon, the PCR reaction was amplified by using the oligonucleotide pair VDT OF and VDT OR. The final volume of the reaction mix was 50 µl, consisted of 50 ng of template DNA, 1X Buffer I-Taq (BioRad Laboratories Inc), 125 µM dNTPs (BioRad Laboratories Inc), 2.0 mM of Magnesium (Mg) (BioRad Laboratories Inc), 200 nM of each primer, and 2.5 U i-Taq DNA polymerase (BioRad Laboratories Inc). The cycling parameters for the amplification reactions were as follow: an initial denaturation step at 95 °C for 5 min was followed by 25 cycles of denaturation steps at 94 °C for 1 min, annealing steps at 68 °C for 2 min and extension at 72 °C for 2 min. PCR product were hold at 16 °C after a final extension at 72 °C for 5 min.

Two microlitre of the PCR reaction was then used to amplify the *P. vivaxdhfr* (*pvdhfr*) domain of size 711 bp by using oligonucleotide pair VDT OF and VDF OR (first PCR). The PCR reaction mixtures were the same as used in the first PCR reaction except for the Mg concentration and the primer concentration. The cycling temperature was also similar to the first PCR amplification except for the annealing temperature and the number of cycles (see Table 1).

**Table 1:** Primers and profiles used for amplification of *pvdhfr* gene

Primer pairs (5′–3′)	Concentration of Mg2 (mM)	Concentration of primer (nM)	Annealing Temperature (°C/Min)	Cycle	Size (bp)
VDT OF: ATGGAGGACCTTTCAGATGTATTTGACATT VDT OR: GGCGGCCATCTCCATGGTTATTTTATCGTG	2.0	200	68/2	25	1876
VDT OF: ATGGAGGACCTTTCAGATGTATTTGACATT VDF OR: CTTGCTGTAAACCAAAAAGTCCA	2.0	400	66/2	30	711
VDF 13NF: GACCTTTCAGATGTATTTGACATTTACGGC VDF NR58: GGTACCTCTCCCTCTTCCACTTTAGCTTCT	1.5	300	62/2	30	232
VDNF57: CATGGAAATGCAACTCCGTCGATATGATGT VDF NR: TCACACGGGTAGGCGCCGTTGATCCTCGTG	1.5	400	50/2	30	472
VDT OF: ATGGAGGACCTTTCAGATGTATTTGACATT VDF NR: TCACACGGGTAGGCGCCGTTGATCCTCGTG	1.0	300	60/2	30	608

Nested PCR were carried out separately to amplify three regions on the *pvdhfr* genes. The PCR reaction used primer pairs VDF13NF and VDFNR58 amplifying 232 base pair (bp) fragment containing Ile13Leu, Pro33Leu, Ser58Arg, and Thr61Met, primer pair using VDNF58and VDF NR amplifying 472 bp fragment containing Phe58Ile/Leu and Ser117Thr/Asn, and primer pair VDTOF and VDFNR amplifying 608 bp fragment containing Phe58Ile/Leu and Ile173Leu. The PCR reaction were similar to the PCR reaction of the first PCR, except for the concentration of Mg and primer concentration. The cycling temperature was also similar to that of the first PCR except for the annealing temperature and the cycles (see Table 1).

Amplification of the *P. vivax dhps* (*pvdhps*) gene was carried out as described previously [19]. The first round PCR was performed by using the primer sets VDHPS OF and VDHPS OR, followed by two sets of second round PCR using primer sets VDHPS NF and VDHPS NR amplifying 703 bp fragment containing codon Ala383Gly, and primer sets VDHPS-553OF and VDHPS NR amplifying 170 bp fragment containing codon 553. The first and second rounds PCR were carried out in a final volume of 50 µl with similar reaction as carried out for *pvdhfr*, except for the primers and Mg concentration and the cycling temperature (see Table 2).

**Table 2:** Primers and profiles used for amplification of *pvdhps* gene

Primer pairs (5′–3′)	Concentration of Mg2 (mM)	Concentration of primer (nM)	Annealing Temperature (°C/Min)	Cycle	Size (bp)
VDHPS OF: ATTCCAGAGTATAAGCACAGCACATTTGAG VDHPS OR: CTAAGGTTGATGTATCCTTGTGAGCACATC	2.0	250	69/2	21	1354
VDHPS NF: AATGGCAAGTGATGGGGCGAGCGTGATTGA VDHPS NR: CAGTCTGCACTCCCCGATGGCCGCGCCACC	2.0	250	70.5/2	25	703
VDHPS 553OF: TTCTCTTTGATGTCGGCCTGGGGTTGGCCA VDHPS NR: CAGTCTGCACTCCCCGATGGCCGCGCCACC	2.0	250	69.5/1	30	170

The PCR products were electrophoresed on 2 % agarose gel (Biorad Laboratories Inc) with GelTed™ Nucleic Acid Gel Stain and visualized on an ultraviolet transilluminator. The lack of cross-contamination was monitored by the inclusion of negative control samples (water was used to replace the DNA template) in each of amplifications carried out.

### RFLP (Restriction fragment length polymorphism) for detection of specific mutations in *pvdhfr* and *pvdhps*

The presence of mutations on *pvdhfr* at codons 13Leu, 33Leu, 57Ile/Leu, 58Arg, 61Met, 117Asn/Thr, and 173Leu and mutation on *pvdhps* at codons 383Gly and 553Gly were analysed by RFLP following protocol as described previously [7]. The enzyme digestions were conducted according to manufacturer’s instruction (New England Biolabs, Beverly, MA, USA). Details of the restriction enzymes used and the digestion product sizes indicating wild type and mutant are described in Table 3. The detection of mutation at 13Leu, 33Leu, 58Arg, 61Met, and 173Leu was determined by *HaeII*, *SacII*, *AluI*, *Tsp451*, and *StyI*, respectively. The detection of mutation at 57Ile/Leu was determined by reaction of PCR (VDT-OF/VDFNR) with *Xmn1*, which did not cleave the 608 bp product. To confirm the mutation, either 57Ile or 57Leu, the PCR (VDNF57/VDF NR) was digested with *BsrGI* to digest the 472 bp products to 444 and 28 bp (mutation 57Ile) and no digestion (mutation 57Leu). The detection of mutation 117Thr/Asn was determined by reaction of PCR (VDNF57/VDF NR) with *PvuII*, which did not digest the 472 bp product. The codon Ser117Thr was determined by reaction with *BsrI*, which did not digest the 472 bp. The mutation at 117Thr was determined by reaction with *BstNI*, which cleaved the 472 bp product to 258 and 215 bp. The mutation at 117Asn was determined by reaction with *BsrI* which cleaved the 472 bp to 253 and 219 bp. The mutation at 117Asn was confirmed by reaction with *BstNI*, which did not cleave the 472 bp product. Amplification of *pvdhps* gene, region VDHPS NF/VDHPS NR, detected mutation at 383Gly. The mutation was detected by reaction of the PCR product with *MspI*, which cleaved the 703 bp product to 655 and 48 bp. The mutation at 553Gly was detected by reaction of PCR VDHPS553OF/VDHPSNR with *MscI*, which did not digest the 170 bp.

**Table 3:** The primer pairs, PCR product sizes, restriction enzymes used and the digestion product size in detection of gene polymorphism on *pvdhfr* and *pvdhps* gene

Gene/primer pair	PCR product size (bp)	To detectmutation at codon	Restriction enzyme used	Digestion product size (bp)	
				Wild type	Mutation
DHFR Forward: VDF 13NF Reserve: VDF NR58	232	Ile13Leu Pro33Leu Ser58Arg Thr61Met	*Hae*II *Sac*II *Alu*I *Tsp451*	Ile: 232 Pro: 138 and 94 Ser: 167, 40 and 25 Thr: 200 and 32	Leu: 200 and 32 Leu: 232 Arg: 207 and 25 Met: 232
DHFR/ Forward: VDNF57 Reserve: VDF NR	472	Phe57Ile/Leu	*Bsr*GI *Pvu*II *BstN*I *BsrI*	Phe: 472 Ser: 258 and 214 Ser: 472 Ser: 472	Ile: 444 and 28 Leu: 472 Asn/Thr: 472 Thr: 257 and 215 Asn: 472 Asn: 253 and 219 Thr: 472
DHFR/ Forward: VDT OF Reverse: VDF NR	608	Phe57Ile/Leu Ile173Leu	*Xmn*I *Sty*I	Phe: 442 and 166 Ile: 472 and 136	Ile/Leu: 608 Leu: 438, 97 and 73
DHPS/ Forward: VDHPS NF Reverse: VDHPS NR	703	Ala383Gly	*Msp*I	Ala: 703	Gly: 655 and 48
DHPS/ Forward: VDHPS553OF Reverse: VDHPSNR	170	Ala553Gly	*Msc*I	Ala: 143 and 27	Gly: 170

### Analysis of PCR–RFLP products using the Agilent 2100 Bioanalyzer

The PCR–RFLP products were analysed using the Agilent 2100 Bioanalyzer and the Agilent DNA 1000 Kit (Agilent Technologies, Molecular Probes Inc, USA). The procedures were conducted according to manufacturer’s instruction. The result was then viewed and analysed using Agilent 2100 software.

## Results

### Sample collection

A total of 619 and 2119 individuals from 23 and 26 sites (villagers, logger camps, road construction workers) in Kalabakan and Kota Marudu, respectively, participated in the study. In Kalabakan, 58 individuals (9.37 %) (95 % [CI] 7.07–11.67) tested positive for malaria of which 26 (4.2 %) (95 % [CI] 2.6–5.8 %) were positive for *P. vivax*. While in Kota Marudu, 52 individuals (2.45 %) (95 % [CI]1.8–3.1 %) tested positive for malaria of which 11 (0.52 %) (95 % [CI] 0.2–0.8 %) were positive for *P. vivax*. Total number of *P. vivax* samples used in the study was 37. The *pvdhfr* and *pvdhps* genes were successfully amplified and RFLP analysis for detection of mutation on the genes was conducted.

### Analysis of mutation on the *pvdhfr* and *pvdhps* genes

**Fig. 2:**
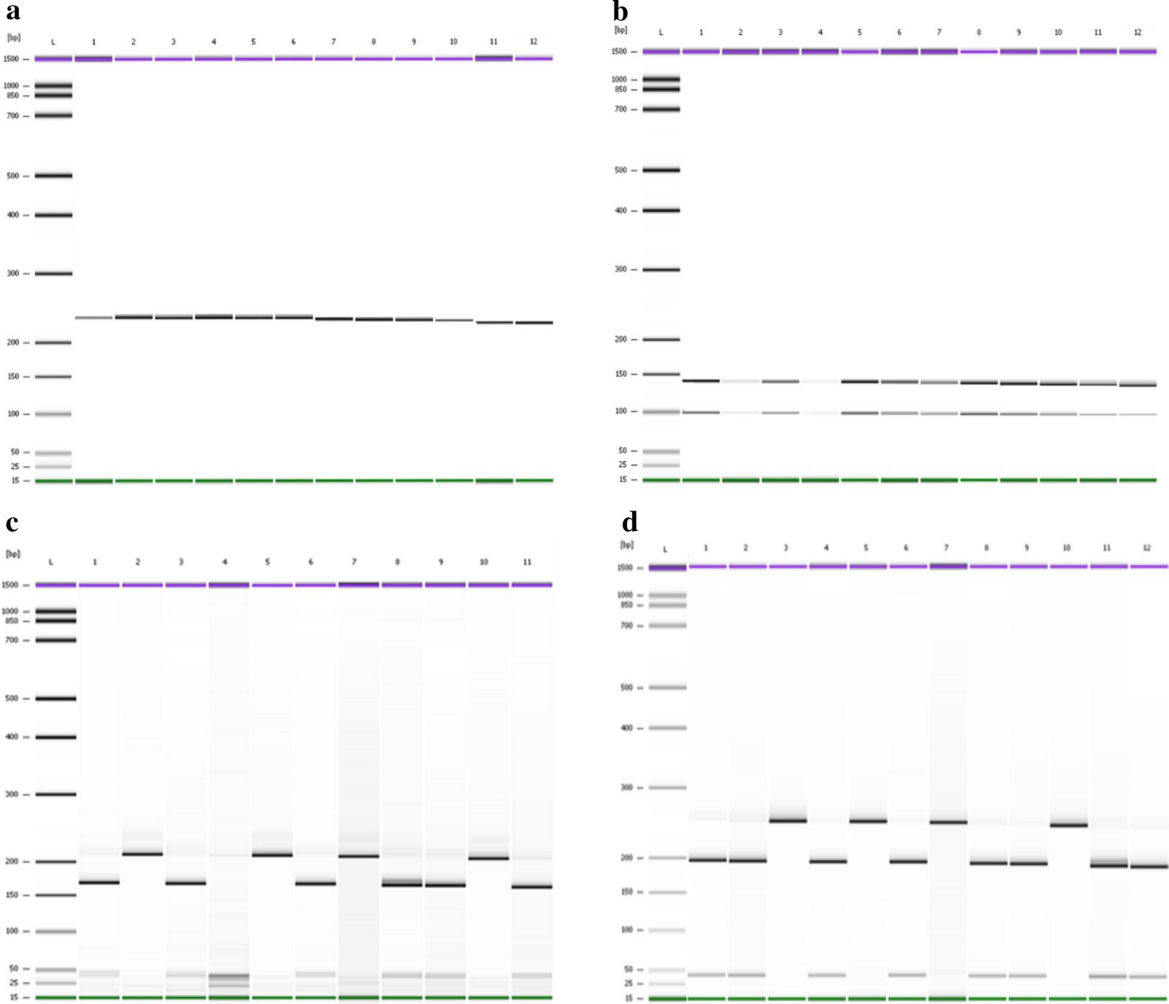
PCR–RFLP of the *pvdhfr* gene. PCR–RFLP products of VDF13NF/VDFNR58 with *HaeIII* showed no cleavage of the 232 bp fragment indicating of wild type Ile13 (**a**). Reaction with *SacII* cleaved the PCR products to 138 and 94 bp indicating wild type Pro33 (**b**). Reaction with *AluI* cleaved the PCR products to 208 and 25 bp fragments, indicating a mutation 58Arg, while cleavage to 167, 40 and 25 bp indicated a wild type Ser58 (**c**). Reaction with *Tsp451* showed no cleavage (232 bp) indicating a mutation 61Met, while wild type Thr61 showed a cleavage to 200 and 32 bp fragment (**d**). **a**, **b** and **d**:* Lane L* DNA ladder of Agilent DNA 1000 Kit (Agilent Technologies, Molecular Probes Inc, USA),* Lanes 1–12* PCR–RFLP products of samples. **c**:* Lanes L* DNA ladder of Agilent DNA 1000 Kit (Agilent Technologies, Molecular Probes Inc, USA),* Lane 1–11* PCR–RFLP products of samples

**Fig. 3:**
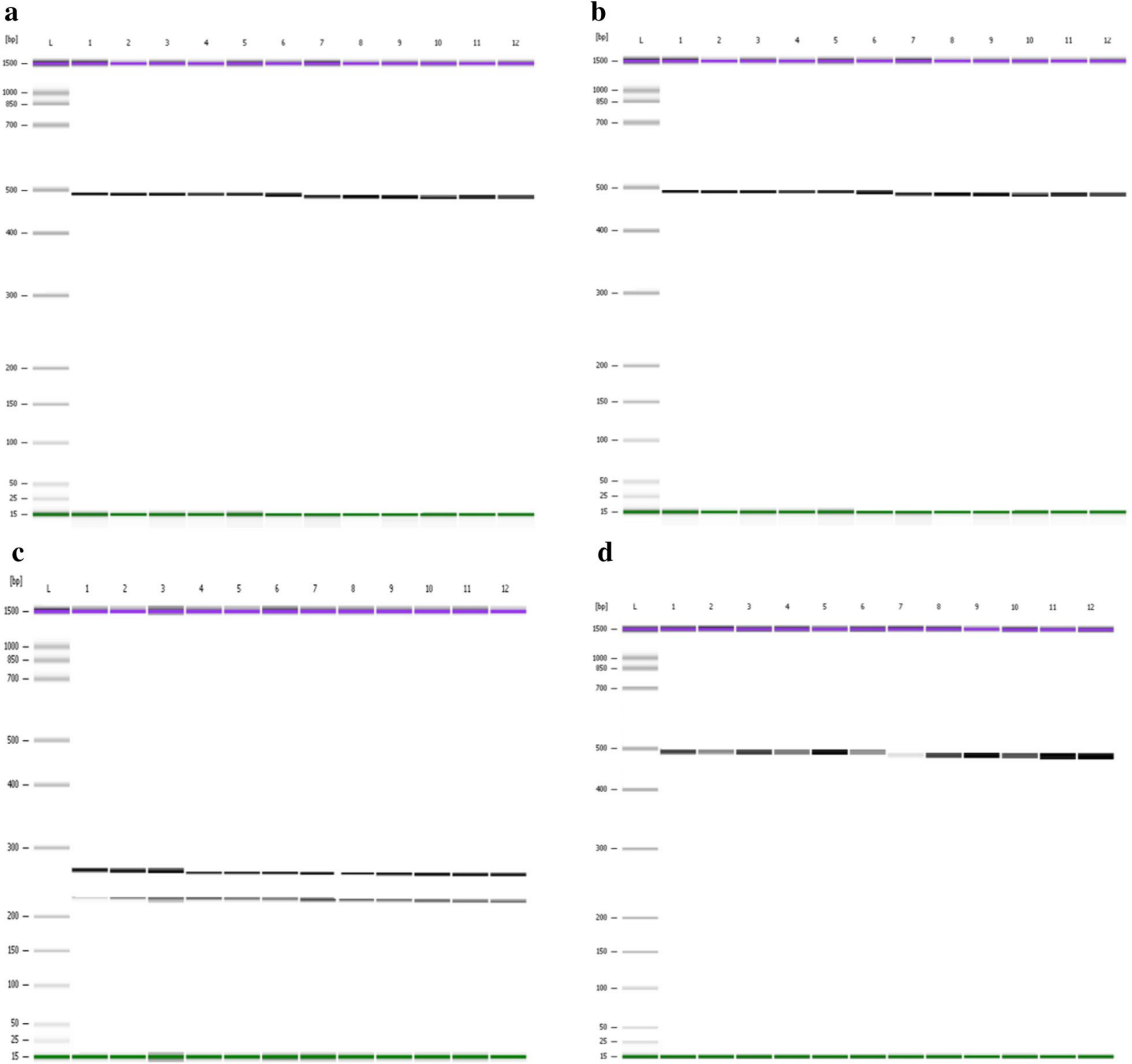
PCR–RFLP of the *pvdhfr* gene. PCR–RFLP product of VDNF 57/VDF NR with *BsrGI* showed no cleavage of the 472 bp indicating wild type Phe58 and mutation 57Leu (**a**). Reaction with *PvuII* showed no cleavage (472 bp) indicating mutation 117Thr/Asn (**b**). Reaction with *BstN1* cleaved the 472 bp to 258 and 215 bp indicating mutation 117Thr (**c**). Reaction with *Bsr1* showed no cleavage indicating wild type Ser118 and mutation 117Thr (**d**). **a**, **b**, **c** and **d**:* Lane L* DNA ladder of Agilent DNA 1000 Kit (Agilent Technologies, Molecular Probes Inc, USA),* Lanes 1–12* PCR–RFLP products of samples

**Fig. 4:**
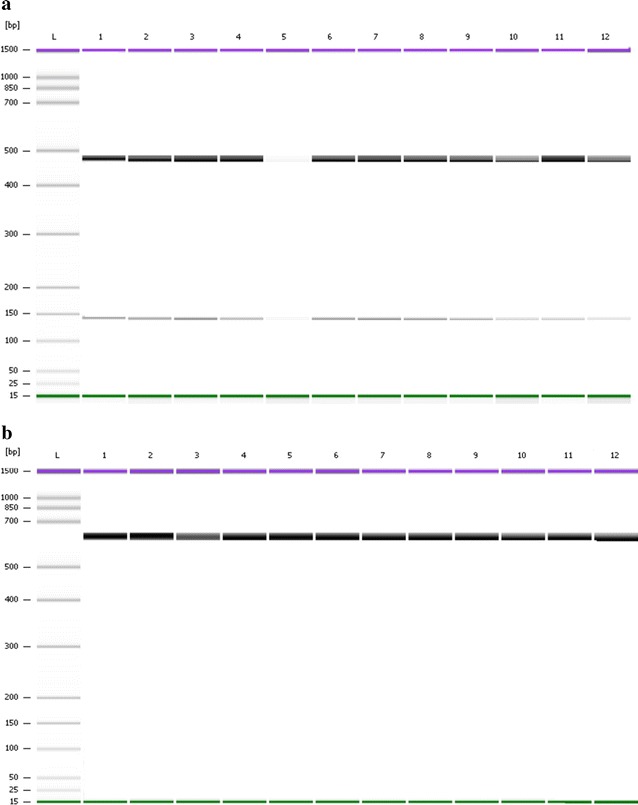
PCR–RFLP of the *pvdhfr* gene. PCR–RFLP of VDTOF/VDFNR with *StyI* cleaved the 608 bp fragment into 472 and 136 bp indicating of wild type Ile173 (**a**). Reaction with *XmnI* showed no cleavage (608 bp) indicating mutation 57Ile/Leu (**b**). **a** and **b**:* Lane L* DNA ladder of Agilent DNA 1000 Kit (Agilent Technologies, Molecular Probes Inc, USA).* Lanes 1–12* PCR-RFLP products of samples

**Fig. 5:**
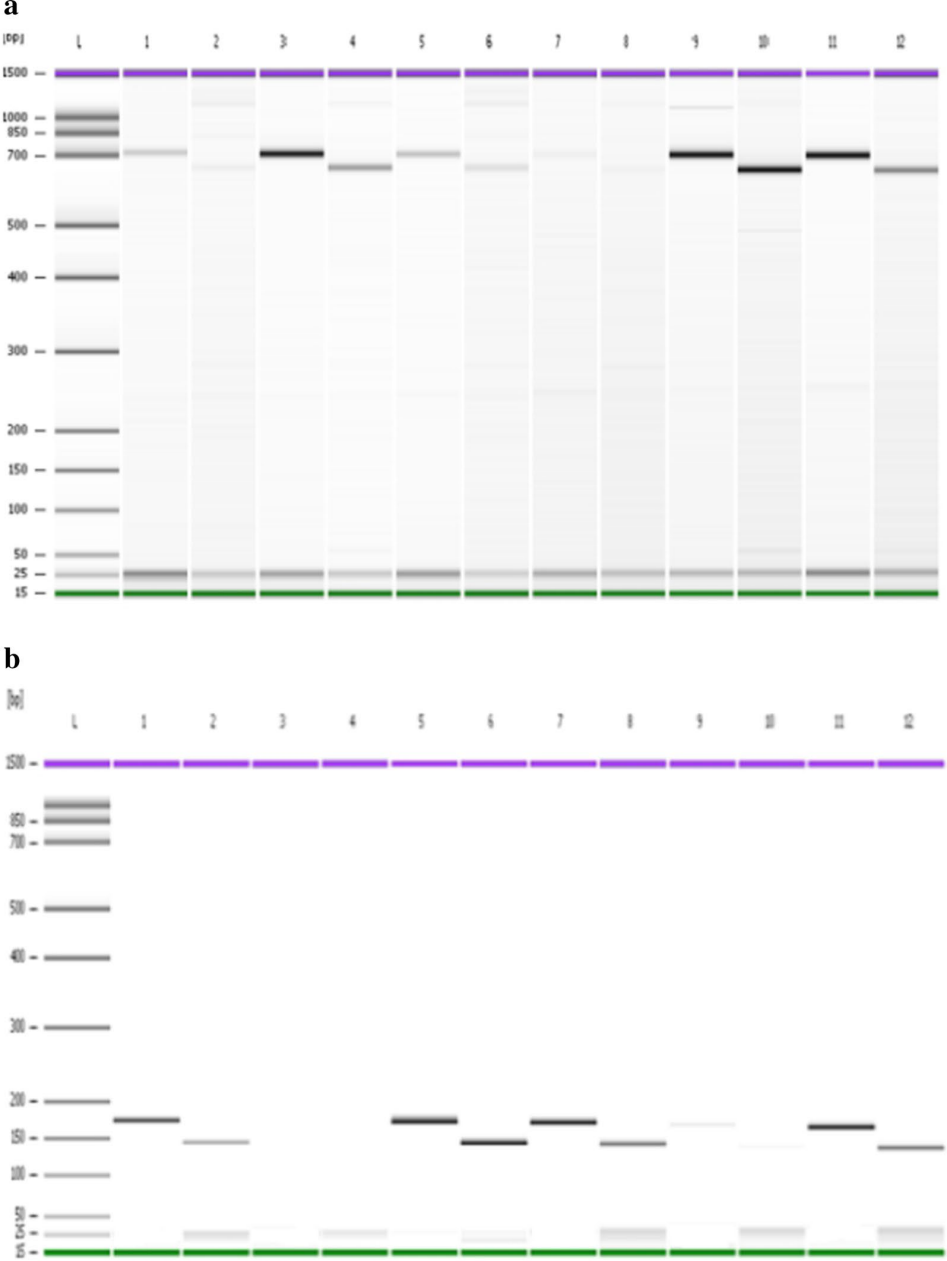
PCR-RFLP of the *pvdhps* gene. PCR-RFLP of VDHPSNF/VDHPSNR with *MspI* cleaved the 703 bp fragment into 655 and 48 bp indicating mutation 383Gly (**a**). PCR–RFLP of VDHPS553OF/VDHPSNR with *MscI* digest the 170 bp to 143 and 28 bp indicating wild type Ala553 (**b**). **a** and **b**:* Lane L* DNA ladder of Agilent DNA 1000 Kit (Agilent Technologies, Molecular Probes Inc, USA). **a**:* Lanes 1, 3, 5, 7, 9, and 11* PCR products of VDHPSNF/VDHPSNR,* Lanes 2, 4, 6, 8, 10, and 12* digestion reaction with *MspI*. **b**:* Lanes 1, 3, 5, 7, 9, and 11* PCR products of VDHPS553OF/VDHPSNR,* Lanes 2, 4, 6, 8, 10, and 12* digestion reaction with *MscI*

Findings of the *pvdhfr* gene showed presence of mutations at 58Arg and 61Met (Fig. 2), at 57Leu and 117Thr (Fig. 3) and 57Leu (Fig. 4). Findings of *pvdhps* gene showed mutation at 383Gly (Fig. 5). Wild type was observed at Ile13, Pro33, Ile173 on *pvdhfr* gene and Ala553 on *pvdhps* gene.The prevalent of wild type and mutant on *pvdhfr* and *pvdhps* genes is summarized in Table 4. No mutation was observed at codon 13, 33 and 173 on *pvdhfr* and at codon 553 on *pvdhps* gene on samples from Kalabakan and Kota Marudu. Common mutations (100 %) on *pvdhfr* were at 57Leu and 117Thr (Table 4). Mutations at 58Arg and 61Met were observed to be higher in Kota Marudu, 72.73 % (95 % [CI] 43.44–90.26). Mutation at 383Gly on *pvdhps* was highest in Kalabakan with 80.77 % (95 % [CI] 62.12–91.49) (Table 4).

**Table 4:** Frequency of wild type and mutation on the *dhfr* and *dhps* gene from 37 *P. vivax* samples collected in Kalabakan and Kota Marudu, Sabah

Genes	Codon	Kalabakan (n = 26)		Kota Marudu (n = 11)	
		Wild type (%)	Mutant (%)	Wild type (%)	Mutant (%)
*Pvdhfr*	13	100	0	100	0
	33	100	0	100	0
	57	0	100	0	100
	58	69.23	30.77	27.27	72.73
	61	69.23	30.77	27.27	72.73
	117	0	100	0	100
	173	100	0	100	0
*pvdhps*	383	19.23	80.77	27.27	72.73
	553	100	0	100	0

### Distribution of *pvdhfr* and *pvdhps* combination alleles

The findings showed that there are four distinct haplotypes of *pvdhfr*/*pvdhps* combination among the 37 samples (Table 5). The four haplotypes are:

**Table 5:** Frequency distribution of the combination *pvdhfr* and *pvdhps* from 37 *P. vivax* samples collected in Kalabakan and Kota Marudu, Sabah demonstrated four distinct haplotypes

*Pvdhfr* alleles	*Pvdhps* alleles	No of isolates (%)		No of *dhfr/dhps* combination mutant genotype
		Kalabakan (n = 26)	Kota Marudu (n = 11)	
13Ile/33Pro/57**Leu**/58**Arg**/61**Met**/117**Thr**/173Ile	383**G**ly553Ala	7 (26.92)	6 (54.55)	4 *dhfr*, 1 *dhps*
13Ile/33Pro/57**Leu**/58Ser/61Thr/117**Thr**/173Ile	383**Gly**/553Ala	14 (53.85)	2 (18.18)	2 *dhfr*, 1 *dhps*
13Ile/33Pro/57**Leu**/58**Arg**/61**Met**/117**Thr**/173Ile	383Ala/553Ala	1 (3.85)	2 (18.18)	4 *dhfr*
13Ile/33Pro/57**Leu**/58Ser/61Thr/117**Thr**/173Ile	383Ala/553Ala	4 (15.38)	1 (9.09)	2 *dhfr*

*Ile* isoleucine, *Leu* leucine, *Ser* serine, *Thr* threonine, *Pro* proline, *Arg* arginine, *Met* methionine, *Ala* alanine, *Gly* glycine

- 13Ile, 33Pro, 57**Leu**, 58**Arg**, 61**Met**, 117**Thr**, 173Ile, 383**Gly**, 553Ala,
- 13Ile, 33Pro, 57**Leu**, 58Ser, 61Thr, 117**Thr**, 173Ile, 383**Gly**, 553Ala,
- 13Ile, 33Pro, 57**Leu**, 58**Arg**, 61**Met**, 117**Thr**, 173Ile, 383Ala, 553Ala and
- 13Ile, 33Pro, 57**Leu**, 58Ser, 61Thr, 117**Thr**, 173Ile, 383Ala, 553Ala

## Discussion

From 2006 to 2012, more than 50 % of malaria cases in Malaysia were attributed by *P. vivax* species, although recently *P. knowlesi* cases has been reported to be prevalent at Lipis Pahang in the Peninsular Malaysia and Sarawak, in Malaysia Borneo [26]. The malaria cases are decreasing; however, cases still occur in confined, remote, interior, hilly or forested areas of endemic regions. Malaria control and elimination strategy plays an important role in decreasing the disease burden in the country [27]. The present study is undertaken to investigate the prevalence of mutation associated with the *pvdhfr* and *pvdhps* in *P. vivax* isolates in the endemic areas of Kalabakan and Kota Marudu in Sabah. In Kalabakan, of the 619 individuals screened for malaria, 9.37 % were positive, of which 4.2 % were *P. vivax* infection, while in Kota Marudu, of the 2119 population screened, 2.45 % positive for malaria, of which 0.52 % were positive for *P. vivax*.

Genotype analysis of the *P. vivax* samples identified that the most prevalent mutations of *pvdhfr* gene from both Kalabakan and Kota Marudu are single mutations at codon 57Leu (100 %) and 117Thr (100 %). Findings from Kuesap et al. [28] in Mae Sot area in Thailand showed a similar observation, where mutation at codon 117Thr was prevalent. Unlike this study, where mutation at 57Leu (100 %) was observed, the finding in Mae Sot, Thailand was found to be the less [28]. Recent studies from Philippine, showed that there are no mutation at acid amino 57 meanwhile all their samples were mutant 117Asn [29].Meanwhile, study from Indonesia showed the allele with the highest prevalence was 117Asn (27 %), followed by 117Thr (18 %) [30]. However,all samples from Kalabakan and Kota Marudu did not contain any 117Asn mutation. Mutation at 57Leu and 117Thr, on its own, confers resistance to pyrimethamine [11, 31] and likely to be precursory to further *dhfr* mutations.

The present study also showed mutation at codon 58Arg (Kalabakan 30.77 % and Kota Marudu 72.73 %) and at codon 61Met (Kalabakan 30.77 % and Kota Marudu 72.73 %). Mutations at 57Leu, 58Arg, 61Met, and 117Thr have been reported to be involved in clinical antifolate resistance [11, 32]. In Kota Marudu, mutation at 58Arg and 61Met was found to be high compared to Kalabakan. However in Kalabakan, a higher proportion of the *P. vivax* populations are still wild type at codon 58Arg and 61Met. Kota Marudu region is nearly with Philippines meanwhile Kalabakan is more nearly to Indonesia. Recent study by from Philippine showed that all their sample represent the mutation at 58 Arg [29] compare with study from Indonesia showed that their sample still have wild type at codon 58 (58Ser) [30]. Studies conducted in the region indicated that mutation at 58Arg is common, similar to other regions, such as East Timor [15], Thailand [25], Philippines [17], and India [31].

Study of the *pvdhps* gene on samples collected from Kalabakan and Kota Marudu showed that mutation at codon 383Gly (80.77 and 72.73 %, respectively) is common. Mutation at codon 383Gly indicated a reduced sensitivity to sulfa drugs and sulfones [14, 19]. Unlike this study, the prevalence of 383Gly mutation is low in other parts of the region, East Timor [33], Iran [34], Pakistan [7], and Korea [16]. Similar with recent study from neighbouring region, Indonesia showed 50 % sample carried mutant allele, 383Gly meanwhile mutant allele 553Gly was not observed in any of the isolates examined [30].

No mutations were detected at codon 13Leu, 33Leu and 173Leu of *pvdhfr* gene and codon 553Gly on the *pvdhps*. Mutation at codon 173Leu was found to be involved in clinical anti-folate resistance [25]. With the presence of 383Gly mutation along with double mutant of *pvdhfr*, which observed in the present study, implicated the use of either pyrimethamine or sulfadoxine (with other anti-malarial drugs) or a combination (SP), in post treatment failure.

In the present findings, there are four distinct haplotypes of *pvdhfr/pvdhps*. The most common combination is Ile13, Pro33, 57**Leu**, Ser58, Thr61, 117**Thr**, Ile173, 383**Gly**, Ala553, (two *dhfr*, one *dhps)* (53.85 %), and was found to be prevalent in Kalabakan. Haplotype Ile13, Pro33, 57**Leu**, 58**Arg**, 61**Met**, 117**Thr**, Ile173, 383**Gly**, Ala553, (four *dhfr*, one *dhps)* was observed in both Kalabakan and Kota Marudu. Clinical studies have showed that quadruple *pvdhfr* mutant parasite (57**Leu**, 58**Arg**, 61**Met**, 117**Thr),** had been associated with SP treatment failure [9, 11, 32]. The mutant parasite in this study, strongly suggested that *P. vivax* isolates from Kalabakan and Kota Marudu carry the resistant allele to antifolate drugs, SP. This observation is also seen in neighbouring countries, Indonesia, Papua New Guinea and India [9, 35].

Mutation combinations at 57Leu, 58Arg with 383Gly and 58Arg, 117Thr with 383Gly, have been implicated in reducing sensitivity to SP drugs in *P. vivax* population [36], and mutation at 58Arg and 117Thr has been implicated in in vivo pyrimethamine resistance as it could cause structural changes in *pvdhfr* and lead to decrease binding to the drug [37], and appeared first under drug pressure [34]. One in vitro study at Indonesia, showed that most of samples that carried the quadruple mutant allele (57Arg/58Leu/61Met/117Thr) were 23 times more likely to experience therapeutic failure, compare with subjects infected by parasites that carried only wild type, single, double or triple mutant allele of *dhfr* [13].

## Conclusion

The study suggests the presence of sulfadoxine, pyrimethamine and SP pressure on *P. vivax* isolates in Kalabakan and Kota Marudu, Sabah. The presence of triple and quintuple mutation combination suggests that *P. vivax* isolates exhibit a high degree of resistance to sulfadoxine and pyrimethamine and SP. The use of parasite molecular markers as tools for surveillance for drug resistance provides support to malaria control programmes and malaria elimination strategies, so that resistance status can be assessed and the most effective treatment can be selected and deployed.

This study served as scientific evidence that there is resistance to sulfadoxine, pyrimethamine and SP in the study area. Any malaria drug combination, such as artesunate plus SP and chloroquine plus SP needs to be reconsidered for the treatment of *P. vivax* infection, especially in the area of chloroquine and sulfadoxine-pyrimethamine-resistant *P. vivax*.
